# Drugs and herbs given to prevent hepatotoxicity of tuberculosis therapy: systematic review of ingredients and evaluation studies

**DOI:** 10.1186/1471-2458-8-365

**Published:** 2008-10-21

**Authors:** Qin Liu, Paul Garner, Yang Wang, Binghua Huang, Helen Smith

**Affiliations:** 1 School of Public Health, Chongqing Medical University, No.1 Yixueyuan Road, Chongqing, PR China; 2 International Health Group, Liverpool School of Tropical Medicine, Liverpool, UK

## Abstract

**Background:**

Drugs to protect the liver are frequently prescribed in some countries as part of treatment for tuberculosis. The biological rationale is not clear, they are expensive and may do harm. We conducted a systematic review to a) describe the ingredients of "liver protection drugs"; and b) compare the evidence base for the policy against international standards.

**Methods:**

We searched international medical databases (MEDLINE, EMBASE, LILACS, CINAHL, Cochrane Central Register of Controlled Trials, and the specialised register of the Cochrane Infectious Diseases Group) and Chinese language databases (CNKI, VIP and WanFang) to April 2007. Our inclusion criteria were research papers that reported evaluating any liver protection drug or drugs for preventing liver damage in people taking anti-tuberculosis treatment. Two authors independently categorised and extracted data, and appraised the stated methods of evaluating their effectiveness.

**Results:**

Eighty five research articles met our inclusion criteria, carried out in China (77), India (2), Russia (4), Ukraine (2). These articles evaluated 30 distinct types of liver protection compounds categorised as herbal preparations, manufactured herbal products, combinations of vitamins and other non-herbal substances and manufactured pharmaceutical preparations. Critical appraisal of these articles showed that all were small, poorly conducted studies, measuring intermediate outcomes. Four trials that were described as randomised controlled trials were small, had short follow up, and did not meet international standards.

**Conclusion:**

There is no reliable evidence to support prescription of drugs or herbs to prevent liver damage in people on tuberculosis treatment.

## Background

China has a long history of traditional medicine, based on the theory of yin yang and its balance with qi (vital energy) [1]. There are over 100,000 defined traditional Chinese medicine therapies and 80% of these are herbal combinations [2]. To treat tuberculosis (TB), traditional Chinese medicine practitioners typically advocate a combination of biomedical treatment to eliminate bacteria, and traditional medicine to strengthen qi [1], and herbs to protect the liver.

Anti-tuberculosis drugs, including isoniazid, rifampicin and pyrizinamide have hepatotoxic effects. A meta-analysis of studies involving several anti-tuberculosis drug regimens estimates the incidence of liver toxicity is 2.6% with co-administered isoniazid and rifampicin, 1.6% with isoniazid alone, and 1.1% with rifampicin alone [3]; some estimates give the incidence of liver damage among people taking anti-tuberculosis drugs in China to be much higher – at around 15–30% of all patients [4,5].

The number of people being prescribing 'liver protection drugs' worldwide is not known, but it is generally agreed that it is almost universal for TB patients in China [6]. These drugs are either given to all patients on anti-tuberculosis treatment, or those with some liver function test abnormalities. There is some emerging evidence that the out of pocket costs to patients are high. For example, a recent descriptive study of TB treatment and policy in one municipality found that on top of the TB drugs (which are free) patients were charged expensive fees for liver protection drugs', and this was an important reason why patients interrupted drug taking [6]. Others report the administration of additional non-TB drugs in China, at a higher than necessary cost to patients [7].

There is no obvious biological rationale for these herbal drugs. Yet clinicians and TB specialists in China appear convinced that these various pharmaceutical and herbal preparations may have a protective effects on the liver in people taking anti-TB treatment. However, a primary concern is patient adverse reactions to herbal drugs; some Chinese herbs are known to cause acute hepatitis, and other herbs used in phytomedicine can cause liver toxicity and even liver failure [2]. It is unclear how the effects, and safety, of these drugs are being evaluated in TB patients.

To help open up the debate in this area, we systematically summarised what these agents are as reported in scientific papers that describe evaluations of these liver protection drugs; and critically appraised the evidence base against international standards for appropriate scientific study designs that could justify their use.

## Methods

### Search

We searched international medical databases (MEDLINE, EMBASE, LILACS, CINAHL, Cochrane Central Register of Controlled Trials, and the specialised register of the Cochrane Infectious Diseases Group) and Chinese language databases (CNKI, VIP and WanFang) to April 2007. In the two step process, we firstly used combinations of key words including: tuberculosis, liver damage, hepatoprotector, liver protection drug. From the literature identified we generated a list of compounds used. In the second step, we searched the databases on the specific names.

### Inclusion criteria and application

We included research papers that reported evaluating any liver protection drug or drugs for preventing liver damage in people taking anti-tuberculosis treatment.

### Data extraction

Two authors (HS, QL) screened titles and abstracts of papers retrieved from English databases; BH and QL screened titles and abstracts of articles retrieved from Chinese databases. For studies written in Chinese, two researchers independently categorised and extracted data from the full text articles. An additional six studies from Russia were included based on the English abstracts; subsequent categorisation and extraction of information on study design was done by a researcher based in Russia who is also fluent in English.

### Analysis

We categorised drugs and herbs using a modified version of WHO standard definitions for the evaluation and research of herbal medicines [8]. Herbal preparations include comminuted or powdered herbal materials, extracts of herbal materials, or preparations made by steeping or heating herbal materials in beverages; finished manufactured herbal products are made from one or more herbs, may contain excipients (non-active substances combined with drugs to make them suitable for administration), but do not include products with chemically defined active ingredients; we defined non-herbal preparations as either combinations of vitamins and other non-herbal substances or pharmaceutical preparations (manufactured drugs).

### Effects

To detect any real effects over and above placebo, and therefore determine the potential value of liver protection drugs would require rigorous scientific testing using a randomised, blinded, controlled trial. We examined the design of each included study against international standards for clinical trials. We looked specifically at whether the studies were prospective, whether patients were allocated randomly to treatment groups, if studies were placebo-controlled, and whether authors defined the length of follow up, or methods used to monitor adverse events.

## Results

We identified 191 research papers (175 from the Chinese databases; 16 from international databases) examining liver protection drugs in TB patients. Overall, 85 research articles reported evaluations of drugs or herbs to prevent drug-induced liver damage in patients taking anti-tuberculosis treatment (we excluded 106 studies of drugs used to treat liver damage). Most were conducted in China (77); other countries were India (2); Russia (4); Ukraine (2).

### a) What are the ingredients?

We categorised the ingredients of drugs and herbs according to the WHO classification of traditional herbal medicines [8]; we added categories to describe drugs derived from non-herbal and other substances (see table 1).

**Table 1:** Categorisation of drugs and herbs by main ingredients

**Type**	**Name**	**Description of ingredients**	**Number of studies**	**Country**
Herbal preparations	Yue hua wan	Various combinations of more than 11 Chinese herbs decocted in water	1	China [10]
	Bu pi yang fei bao gan decoction	11 herbs decocted in water	1	China [12]
	Phytotherapy	Infusions of a combination of herbs (on average 25) tailored to individual patients	1	Russia [13]
Finished manufactured herbal products	Hu gan pian	Tablet including a combination of six ingredients (herbs, animal extract, green bean)	8	China [14-21]
	Silymarin	Extract of milk thistle	11	China [22-32]
	Astragalus	Plant extract given intravenously	1	China [33]
	Astragalus dasyanthus	Tincture containing: coumarins; ferrocoumarins; tanning substances; sugars; saponins; ascorbic acid; flavonoids; phytosterols; tocopherols	1	Russia [34]
	Biphenyldicarboxylate	Tablet with extract of wu wei zi plant	2	China [35,36]
	Biphenyldicarboxylate	Extract of wu wei zi combined with glucurolactone	1	China [37]
	Wu zhi capsule	Capsules with extract of the fruit of the wu wei zi plant	3	China [38-40]
	San shen bao gan capsule	Capsules containing combination of 13 herbs	1	China [41]
	Hugan yi fei granule	Granule containing a combination of 12 herbs	1	China [42]
	Oleanolic acid	Tablet with extract of Swertia herb	5	China [43-47]
	Hugan yao	Exact of Swertia herb combined with inosine and Vit C	1	China [48]
	Dan shen	Injectable preparation of Chinese sage (Salvia Miltiorrhiza Bunge)	3	China [49-51]
	Stimuliv	Tablets containing a combination of: Andrographis Paniculata; Eclipta alba; Peumas Boldus Molina; Cynara scolymus; Fumaria perviflora	1	India [52]
	Galstena	Chelidonium majus; Taraxacum officinale; Carduus crispus (curled thistle extracted with enthanol)	1	Russia [53]
	Optiliv	Capsules containing extracts of: Andrographis paniculata; Picrorhiza kurroa; Eclipta alba; Boerhaavia diffusia; Azadirachta indica; Swetia chirata; Solanum nigrum; Terminalia arjuna; Aphanamixis rohituka; Terminalia chebula; Furmaria indica; Excipients	1	India [11]
	Glycyrrhizin	Capsules or injection containing extract of liquorice (gan cao)	6	China [54-59]
	Anisodamine	Tablet containing plant extract	1	China [60]
	Spirin or Spirulina	Derived from blue-green algae, contains proteins, vitamins and essential amino acids	2	China [61,62]
			1	Ukraine [63]
Combinations of vitamins and other non-herbal substances	Tiopronin (liu pu luo ning or kai xi lai)	Ramification of Glycine (amino acid)	17	China [64-80]
	Glutathione (GSH) (tai te or a tuo mo lan)	Injectable antioxidant	5	China [81-85]
	ATP	Adenosine Triphosphate	1	China [86]
	Inosine	Inosine combined with vitamin C	1	China [87]
	Essentiale	Vitamin B6, vitamin B12, derivatives of vitamin B3 (nicotinamide), vitamin E and essential phospholipids	1	China [88]
	Gan ning pian	Liver hydrolysate, compounds of vitamin B, inositol, vitamin B6 and vitamin B12	1	China [89]
	Tocopherol acetate	Vitamin E	1	Ukraine [90]
Pharmaceutical preparations	Glucurolactone	Manufactured drug	3	China [91-93]
	Zixoryn	Manufactured drug	1	Russia [94]

Three studies examined herbal preparations for preventing liver damage in TB patients. In two studies from China, the preparations contained 11 different herbs; in one study different combinations of herbs were administered to participants depending on their vital energy (qi) and bodily balance (yin yang). One study from Russia reported the effect of herbal infusions containing on average 25 different herbs tailored to individual patient needs.

51 studies evaluated manufactured herbal products. Extract of milk thistle (Silymarin) was the most frequently evaluated in the studies we identified (9 studies from China). Oleanic acid (extract of Swertia) and glycyrrhizin (extract of Liquorice) were also common in studies conducted in China. Two studies in India evaluated combinations of many different indigenous herbs and plants; one trial examined Stimuliv tablets, the other a capsule called Optiliv.

We found 27 reports of the hepatoprotective effect of combinations of vitamins and other non-herbal substances in TB patients. The most commonly evaluated was Tiopronin, a ramification of glycine (17 studies in China); three studies used the drug named 'liu pu luo ning', the other 14 were of the drug 'kai xi lai'. The injectable antioxidant glutathione (GSH) was also commonly used in studies conducted in China; two different trade names of this drug were evaluated, 'tai te' and 'a tuo mo lan'.

Four studies reported on the effect of manufactured pharmaceutical preparations. Three studies conducted in China concerned Glucurolactone. One study from Russia concerned the drug Zixoryn.

### b) What is the evidence base for their use?

Table 2 summarises the design features of studies evaluating liver protection drugs grouped into those conducted in China, and those carried out in other countries (India, Russia and Ukraine). From the Chinese studies, we identified 51 prospective studies, where TB patients were followed forward in time and prior to the occurrence of liver damage. In most of these studies exact recruitment and follow up times were not clearly stated. All eight studies conducted in other countries were classified as prospective, but again recruitment and follow up times were not clearly stated.

**Table 2:** Key design features of studies evaluating liver protection drugs

Study characteristics	Studies conducted in China (n = 77)	Studies conducted in other countries (n = 8)
Length of follow up		
<1 month	5	1
<6 months	19	4
> or = 6 months	31	3
Unclear	22	0
Clearly prospective		
Prospective	51	8
Retrospective	18	0
Unclear	8	0
Random allocation		
Clear	3	1
No	20	6
Quasi	15	0
Unclear	39	1
Comparisons		
Placebo	0	2
Anti-TB drugs alone	32	2
Alternative liver protection drug(s)	42	4
No	3	
Methods for monitoring adverse events stated	0	6
Adverse events reported	29	2

No studies conducted in China were placebo controlled; most compared one liver protection drug with another or anti-tuberculosis treatment alone. Most studies conducted in other countries were open trials, comparing one drug to another with no placebo.

It is interesting to note that no studies in China described the methods for monitoring for adverse events, and more than half did not report adverse events at all. Six out of eight non-Chinese papers provided methods for monitoring adverse events.

The length of follow up reported in the Chinese studies varied from less than a month, to more than six months; in 22 studies the exact length of follow up was unclear. The main outcomes assessed in the included studies were indicators of drug-induced liver damage measured by liver function test, such as ALT and AST.

#### Reported as randomised

Overall we identified five studies that reported to be randomised. Three studies conducted in China mentioned randomly allocating patients to treatment groups using random number tables. Two studies conducted in India mentioned randomisation, but only one stated using random number tables to allocate patients. Methods to conceal allocation were not described in the studies from China; one of the Indian studies described concealing the drug codes in a sealed envelope. Two of the studies conducted in China followed patients for two months or less; the other did not specify a follow-up period. The study in India that was clearly randomised followed patients for less than 2 months (45 days), and the study where randomisation was unclear followed patients for two months. All three Chinese studies included less than 250 participants (one included only 80); both the Indian studies included less than 150 participants.

## Discussion

This is the first attempt that we are aware of to systematically summarise the ingredients of drugs and herbs used to protect the liver in patients taking anti-tuberculosis treatment, and to scrutinise the methods used to evaluate their effects. The literature suggests a large difference in the proportion of patients with hepatotoxicity in China (15–30%) compared to elsewhere (about 2.6%). We believe this is due to varying definitions and understanding of what hepatoxicity is, with a more liberal interpretation in the Chinese context. However, we have not systematically examined the literature to clarify this.

Our study revealed thirty distinct types of liver protection drugs or preparations used in patients with TB, and each contains a complex combination of herbal extracts, vitamins or compounds. Few studies provided a clear biological or clinical rationale for the use of specific herbs or drugs, but we know that some of the herbs identified in our summary have already been studied in China with researchers claiming general hepatoprotective properties, although not in TB patients. For example, Silymarin is known to be widely used as a remedy for liver diseases and glycyrrhizin has been used in chronic viral hepatitis [2,9]. The numerous ingredients of Chinese herbs and drugs make it more difficult to determine the active ingredients or properties of these preparations or drugs, something we recognised when extracting information from our included studies. It seems unconvincing that so many different preparations all have properties that protect against liver damage by drugs used to treat TB, and we do not know of any convincing biological mechanism. In addition, we do not know the number of studies that were sponsored by pharmaceutical companies as this is not generally reported in Chinese research papers.

Relatively few studies we identified evaluated herbal preparations; most were concerned with the effect of finished manufactured herbal products. Herbal preparations are more complicated to evaluate, and it has been suggested that a conventional randomised controlled and blinded trial design might not be appropriate [8]. Preparations usually have a distinct smell or taste and the herbs used are sometimes blended to suit individual patient needs; all of which preclude blinding of participants and trialists, and use of placebo preparations. For example, one study of the Chinese herbal preparation 'yue hua wan' claimed to be a prospective study that used a random number table to allocate patients to standard anti-tuberculosis treatment plus the herbal preparation or anti-tuberculosis treatment alone [10]. The study is weakened by the lack of a placebo control, but the published report does not describe the preparation in detail, so we cannot determine whether a placebo preparation would have been possible. Studies that fail to use a placebo-control cannot determine the effect of the herb or drug relative to a control treatment and this is less useful for clinical decision making.

Our summary indicates that the evidence base for the use of liver protection drugs in TB patients comprises mainly small, poorly conducted studies that do not reach the standards of trials used in reliable systematic reviews such as those produced by the Cochrane collaboration. Only one study, out of 85 we identified in the international literature, appeared to be a prospective, randomised, placebo controlled trial of a manufactured herbal product [11]. But the trial followed patients for 45 days only, too short a time period to identify liver damage or toxicity; the authors failed to specify particular outcomes a priori; and the study included only 70 patients.

Sample size is a critical consideration in relation to the primary outcome in studies evaluating liver protection drugs-liver damage or toxicity. We have estimated that to detect an effect on an incidence of liver damage of 15–30% in China [4,5] would require a sample size of 1145 for each trial arm with 95% confidence and 90% power, and if the calculation is based on a global incidence of 1–2% [3], a much larger sample size would be required. It would be impractical and expensive to run trials of this size, and trials of these drugs are not really justified.

Although we classified most of the studies we identified from the Chinese literature as 'prospective', only three appeared to randomly allocate participants to groups, and these were not placebo controlled. Eighteen studies appeared to be retrospective, and used patient records to examine outcomes historically. Generally we found it difficult to determine from the short full text journal reports which particular observational design the authors had followed. Most studies classified as retrospective appeared to be case reports or case series. The reports generally did not describe the research procedures in detail, and it was difficult to determine which studies were case-controlled.

An important finding is the lack of attention in the research papers to adverse events associated with the drugs or herbs used. Some studies mentioned adverse events, but did not describe methods used routinely to monitor patients for events; a large number of the Chinese studies did not mention adverse events at all. Given the existing concerns over the safety of some herbs in use [2], it is important that studies include methods to routinely assess participants for adverse events; that samples are large enough; and studies follow patients over a sufficient period of time to detect infrequent events.

Other authors report poor methodological quality of studies evaluating herbal preparations; often studies do not adequately randomise participants, do not specify outcome measures or end points, contain small sample sizes, and suffer from publication bias [2]. WHO provide guidelines for good practice in conducting clinical trials of herbal medicine, and recommend the design of trials be adapted to the peculiarities of herbal medicines [8].

## Conclusion

In terms of policy implications, this review indicates there is no reliable evidence to support taking liver protection drugs with TB treatment. Indeed some may do harm. Thus there is no medical reason to continue prescribing them; and, given the wide variety of compounds, many of which may have side-effects, it is more likely that they will do more harm than good. Understanding of what liver toxicity is and criteria for diagnosis are probably also different which further complicates any comparison.

In terms of research, it could be argued that trials following accepted international standards for conduct and reporting are required. However, given the poor rationale for using the drugs and large sample size required, then the reasons for doing a proper RCT will be limited. The one justification could relate to the fact that they are widely prescribed, so identifying no effect, or a harmful effect, could potentially be a powerful influence on current policies.

## Competing interests

The authors declare that they have no competing interests.

## Authors' contributions

HS and QL conceived of the study and designed the research with input from PG and YW. HS, BH and QL searched and retrieved studies. HS and QL screened papers in English for inclusion; BH and QL screened papers in Chinese. HS and QL extracted, categorised and interpreted data and drafted the manuscript. PG and YW helped revise the manuscript, and all authors read an approved the final version.

## Pre-publication history

The pre-publication history for this paper can be accessed here:


